# KIRREL promotes the proliferation of gastric cancer cells and angiogenesis through the PI3K/AKT/mTOR pathway

**DOI:** 10.1111/jcmm.18020

**Published:** 2023-11-01

**Authors:** Tao Wang, Shuo Chen, Ziliang Wang, Siyu Li, Xichang Fei, Tong Wang, Mingjun Zhang

**Affiliations:** ^1^ Department of Oncology The Second Affiliated Hospital of Anhui Medical University Hefei Anhui China; ^2^ Department of General Practice The Second Affiliated Hospital of Anhui Medical University Hefei Anhui China

**Keywords:** angiogenesis, gastric cancer, KIRREL, PI3K/AKT/mTOR, targeted therapy

## Abstract

Anti‐angiogenesis is a promising therapeutic strategy for delaying tumour progression that offers, new hope for gastric cancer targeted therapy. The purpose of this study was to investigate the precise mechanism by which Kin of IRRE‐like protein 1 (KIRREL) contributes to the development of gastric cancer, particularly in terms of tumour angiogenesis. Differential expression of KIRREL in tissues and cells was detected using quantitative real‐time polymerase chain reaction, western blotting and immunohistochemistry. A bioinformatics analysis was conducted to screen for the function and pathway enrichment of KIRREL in gastric cancer. Lentivirus‐induced KIRREL silencing in SNU‐5 cells and lentivirus‐induced KIRREL overexpression in AGS cells were used to study the effect of KIRREL on the proliferation, cell cycle and angiogenesis of gastric cancer cells. Moreover, the expressions of PI3K, P‐PI3K, AKT, P‐AKT, mTOR, P‐mTOR, HIF‐1α and VEGF were also detected. Gastric cancer tissues and cells had high levels of KIRREL expression, which is associated with the proliferation, cell cycle and angiogenesis of gastric cancer cells. After silencing and overexpressing KIRREL in SNU‐5 and AGS cells, respectively, the proliferation and angiogenesis of SNU‐5 cells were inhibited, while the proliferation and angiogenesis of AGS cells were promoted. According to a bioinformatics analysis of the KIRREL gene, angiogenesis regulation and the PI3K/AKT pathway were highly connected. The PI3K/AKT/mTOR pathway was repressed and stimulated by KIRREL silencing and overexpression, respectively. IGF‐1, an AKT agonist, and LY294002, an inhibitor, reversed the effects of KIRREL silencing and overexpression on the PI3K/AKT/mTOR pathway and on gastric cancer cell proliferation and angiogenesis. KIRREL may mediate the proliferation and angiogenesis of gastric cancer cells through the PI3K/AKT/mTOR signalling pathway. These findings could help in the further development of potential anti‐angiogenesis targets.

## 1 INTRODUCTION

Gastric cancer (GC) is one of the most common malignant cancers in the world. GLOBOCAN estimates that there will be approximately 1,089,103 new cases and 768,793 deaths from GC in 2020, meaning it ranks, fifth (5.6%) and fourth (7.7%) among the 36 global cancers.^1^ GC is the second most common tumour and the second leading cause of cancer‐related deaths in China.^2^ The absence of distinct symptoms in early GC has contributed to the poor detection rate. As a result, most patients have already missed their opportunity for surgery on the initial visit. In addition, owing to the high incidence, metastasis rate, and mortality rate and low radical resection rate,^3^ the overall prognosis of GC remains poor, especially for advanced GC, where the 5‐year survival rate is only approximately 10%.^4^, ^5^ At present, the main treatment methods for advanced GC are chemotherapy, radiotherapy, molecular‐targeted therapy and immunotherapy.^6^, ^7^ With the rapid development of molecular biology research in cancer treatment, many targeted drugs have been shown to exhibit anti‐tumour activity in GC and to improve patient prognosis. Examples include the epidermal growth factor receptor tyrosine kinase inhibitor cetuximab,^8^ vascular endothelial growth factor (VEGF) inhibitors bevacizumab^9^ and ramucirumab,^10^ and multi‐target anti‐tumour drugs sorafenib^11^ and sunitinib.^12^ In addition, trastuzumab is recommended for HER2‐positive patients with GC.^13^ Conventional chemotherapy regimens combined with molecular‐targeted therapy often have a higher response rate than monotherapy and thus offer better, survival rates to patients.^7^ However, acquired drug resistance is an enormous challenge for targeted therapy^14^ sand has a serious impact on the therapeutic effect. Therefore, the development of novel molecular biomarkers is of great practical significance and provides a new opportunity for the comprehensive treatment of GC.

Tumour growth and distant metastases depend on new capillaries for the delivery of essential nutrients and oxygen.^15^, ^16^ As early as 1971, Folkman proposed the hypothesis of ‘anti‐angiogenic’ therapy for tumours,^17^ which led to extensive research and development in this field. It has been established that the downstream effects of angiogenesis can be therapeutically blocked, inhibiting the growth of capillaries and placing tumours in a ‘dormant’ state.^18^, ^19^ This was made possible by the discovery of the VEGF family and the subsequent development of drugs targeting VEGF. In recent years, therapeutic methods targeting inhibition of the VEGF pathway have achieved good results in clinical trials for several advanced cancers, including colorectal,^20^ lung,^21^ liver,^22^ kidney^23^ and urothelial cancers.^24^ In 2004, bevacizumab, the first anti‐angiogenic drug, was approved by the FDA as a first‐line treatment for metastatic colorectal cancer.^25^ Subsequently, other anti‐angiogenic agents, such as ramucirumab and fruquintinib, have been reported to improve overall survival in patients with gastrointestinal malignancies.^26^, ^27^, ^28^, ^29^ Several new approaches to this pathway have been developed. Anti‐angiogenic therapy therefore appears to be a promising option for GC treatment.

Kin of IRRE‐like protein 1 (KIRREL) is a member of the immunoglobulin superfamily, commonly known as NEPH1.^30^ Previous studies have detected KIRREL expression in the human renal cortex, as well as in the mouse heart, liver smooth muscle, lung, kidney and brain.^31^, ^32^, ^33^ As a main component of the renal diaphragm, KIRREL plays an important role in maintaining the cytoskeleton of renal epithelial podocyte actin and glomerular filtration function.^34^, ^35^ Huber et al. also reported that KIRREL interacts with the P85 subunit of phosphatidylinositol 3‐kinase (PI3K), resulting in an increase in AKT activity and a decrease in cell death induced by apoptotic stimulation.^36^ To date, mainstream research on KIRREL has focused on kidney‐related diseases. Although several recent bioinformatics analyses have found that KIRREL is abnormally expressed in breast cancer and melanoma and is closely related to patient prognosis,^37^, ^38^ the specific mechanism of action has not been reported to date. In previous studies, we identified the differential expression of KIRREL in human GC tissues and normal gastric tissues through bioinformatics analysis and concluded that KIRREL is overexpressed in GC tissues and is closely associated with poor prognosis through immunohistochemical verification and survival analysis.^39^ Based on these results, we further explored the mechanism of KIRREL in GC in this study. We demonstrate the key role of KIRREL in tumour progression and speculate that it might promote tumour cell proliferation and angiogenesis by activating the PI3K/AKT/mTOR pathway, revealing KIRREL as a potential target for GC therapy.

## 2 MATERIALS AND METHODS

### 2.1 Patients and specimens

GC tissues and adjacent tissues (more than 5 cm away from the tumour boundary) from 10 patients were surgically resected in the Department of General Surgery at the Second Affiliated Hospital of Anhui Medical University and stored in a refrigerator at −80°C. All patients were diagnosed with GC based on histopathology, and those with a history of other malignancies were excluded. The clinical information of the patients is presented in Table 1. The study was approved by the Ethics Review Board of the Second Affiliated Hospital of Anhui Medical University (YX2022‐075(F1)) and conducted in accordance with the Declaration of Helsinki. Written informed consent was obtained from all the patients.

**TABLE 1:** Clinical information of 10 GC patients.

Characteristics	Number
Number	10
Gender	
Female	1
Male	9
Age	
≤65	5
>65	5
Pathologic T stage	
T1 & T2	0
T4 & T3	10
Pathologic N stage	
N0 & N1	4
N2 & N3	6
Pathologic M stage	
M0	2
M1	8
Recrudesce	
Yes	3
No	7

### 2.2 Cell culture and transfection

The human cell line GES‐1, GC cell lines SGC7901, SNU‐5, AGS and human umbilical vein endothelial cells (HUVECs) were purchased from the BeNa Culture Collection (Henan, China). GES‐1, SGC7901 and SNU‐5 were cultured in RPMI‐1640 medium (Basalmedia, China), AGS was cultured in Ham's F‐12 medium (Procell, China), and HUVECs were cultured in DMEM (Basalmedia, China). The culture medium contained 10% foetal bovine serum (Gibco, USA), penicillin and streptomycin (100 U/mL, Beyotime, China) and was incubated at 37°C in a 5% CO_2_ incubator. GES‐1 is immortalized gastric normal mucosal cells, AGS is gastric adenocarcinoma cells taken from primary foci, and SGC7901 is gastric adenocarcinoma cells taken from metastatic foci, all of which are adherent to the wall.SNU‐5 is gastric carcinoma cells taken from ascites that are grown in suspension.

The lentiviral vectors containing SH‐NC, SH‐1, SH‐2 and SH‐3 (ZHBY Biotech, China) were transfected into SNU‐5 cells in the logarithmic growth phase (SH‐1: 5′‐GCTCAACTACTCTGGAATTGT‐3′, SH‐2: 5′‐GCACCAATGTCAGCACTTTAG‐3′ and SH‐3: 5′‐GCTGTCCTACGAGAACTATGA‐3′) to construct KIRREL gene silenced cells. In addition, the lentiviral vector carrying the KIRREL sequence (ZHBY Biotech, China) was transfected into AGS cells in the logarithmic growth phase (forward: 5′‐CGCAAATGGGCGGTAGGCGTG‐3′ and reverse: 5′‐AGTCCCGTCCTAAAATGTC‐3′) and used to construct KIRREL‐overexpressing cells.

### 2.3 Immunohistochemistry (IHC)

After roasting at 65°C for 2 h, the slides containing the tissue were dewaxed in xylene for 20 min. Subsequently, the slides were hydrated using an ethanol solution and pure water and placed in a citrate buffer at a high temperature and pressure for 2 min. After 90% methanol and 3% hydrogen peroxide solution were used to remove endogenous peroxidase, the slides were blocked in 5% bovine serum albumin at room temperature for 30 min then incubated with primary antibody at 4°C for 8 h and secondary antibody at room temperature for 30 min. Finally, a diaminobenzidine (DAB) kit was used for colour development (CWBIO, China), and haematoxylin was counterstained and observed under a microscope. The following diluted antibodies were used according to the instructions: KIRREL (BS‐6435R, Bioss, China), PI3K (20584‐1‐AP, Proteintech, China), P‐PI3K (AF3241, Affinity, China), mTOR (66888‐1‐Ig, Proteintech, China), P‐mTOR (ab109268, Abcam, UK), AKT (60203‐2‐Ig, Proteintech, China), P‐AKT (66444‐1‐Ig, Proteintech, China), HIF‐1α (20960‐1‐AP, Proteintech, China) and VEGF (19003‐1‐AP, Proteintech, China).

### 2.4 CCK‐8 assay

The CCK‐8 cell proliferation assay kit (KeyGEN BioTECH, China) was used to analyse cell proliferation ability according to the manufacturer's instructions. The cell concentration was adjusted to 5 × 10^3^ cells/well, the cells were seeded into 96‐well plates (100 μL/well), and CCK‐8 reagent (10 μL/well) was added after incubation for 24, 48 and 72 h, respectively. After 1 h of treatment, absorbance at 450 nm was measured using an automatic microplate reader.

### 2.5 Flow cytometry assay

The cells of each Group were digested and centrifuged, and the supernatant was discarded and washed with phosphate‐buffered saline (PBS), and then, the PBS was discarded. Finally, 1 mL of DNA staining solution and 10 μL of permeabilization solution (MultiSciences Biotech, China) were added, and the cells were oscillated for 5–10 s using a vortex oscillator and incubated for 30 min at room temperature in the dark. The assay was then performed using a flow cytometer (ACEA Biosciences, USA).

### 2.6 Tube formation assay

Matrigel (CORNING, USA), melted at 4°C, was spread neatly tiled in pre‐cooled 24‐well plates (250 μL/well) and placed in a cell incubator to solidify for 30 min. The HUVECs were resuspended in the supernatant of the AGS and SNU‐5 cell lines and then seeded into 24‐well plates containing matrix gel (8 × 10^4^ cells/well) for 6 h for further culture. Angiogenesis was recorded, and the total vessel branch length was analysed using ImageJ software.

### 2.7 Quantitative real‐time PCR (qPCR) assay

TRIzol Reagent (CWBIO, China), ultrapure RNA (CWBIO, China) and SYBR qPCR Master Mix (Vazyme, China) were used according to the manufacturer's instructions and HiScript II Q RT SuperMix for qPCR (+gDNA Wiper) (Vazyme, China) for cell and tissue lysis, total RNA extraction, cDNA synthesis and qRT‐PCR. The primers were designed as follows: KIRREL (F: 5′‐GAGTATGAGATGAAGGACCCCA‐3′ and R: 5′‐GGGCACGGTAGTCAGCATAG‐3′) and β‐actin (F: 5′‐TGGCACCCAGCACAATGAA‐3′ and R: 5′‐CTAAGTCATAGTCCGCCTAGAAGCA‐3′).

### 2.8 Western blotting (WB) assay

Total protein was extracted from the gastric tissue and SNU‐5 and AGS cell lines using conventional methods. Equal amounts of protein samples (15 μg each) and molecular weight markers (Thermo Fisher, USA) were separated using sodium dodecyl sulfate‐polyacrylamide gel electrophoresis (SDS‐PAGE) and electrically transferred to a poly (vinylidene fluoride) membrane, which was then blocked in 5% skim milk for 2 h and incubated with the primary antibody overnight at 4°C. After incubation with secondary antibodies for 2 h at 25°C, protein bands were visualized using an enhanced chemiluminescence detection kit (Thermo Fisher, USA). They were also washed appropriately between each step using Tris‐buffered saline containing 0.1% Tween‐20 (TRIS‐buffered saline containing 0.1% Tween‐20). The following antibodies were used: KIRREL (BS‐6435R, Bioss, China), PI3K (AF6241, Affinity, China), P‐PI3K (AF3242, Affinity, China), AKT (AF6261, Affinity, China), P‐AKT (AF0832, Affinity, China), m‐TOR (AF6308, Affinity, China), P‐mTOR (AF3308, Affinity, China), HIF‐1α (AF1009, Affinity, China), VEGF (AF5131, Affinity, China) and GAPDH (TA‐08, ZSGB‐BIO, China).

### 2.9 Bioinformatics analysis

The gene expression data from the 375 GC samples and 32 normal samples were obtained from the TCGA database (https://portal.gdc.cancer.gov/). The R package ‘stat’ was used for gene correlation analysis, and the R package ‘clusterProfiler’ was used for Gene Ontology (GO) functional enrichment analysis and Kyoto Encyclopaedia of Genes and Genomes (KEGG) pathway enrichment analysis. The following thresholds were used to filter the enriched pathways: *p*‐adjust <0.05 and *q*‐value <0.2. The GEPIA database (http://gepia.cancer‐pku.cn/) was used to analyse the correlation between KIRREL and the key molecules of the PI3K/AKT pathway.

### 2.10 Statistical analysis

Statistical analyses were performed using the GraphPad Prism 9.0.0 programme. All experiments were repeated three times. The quantitative results are expressed as the mean ± standard deviation. For quantitative comparisons between two groups, the independent sample t‐test was used, while the one‐way analysis of variance was used for comparisons between several groups. The test level was set at *α* = 0.05.

## 3 RESULTS

### 3.1 Expression of KIRREL was increased in GC tissues

**FIGURE 1:**
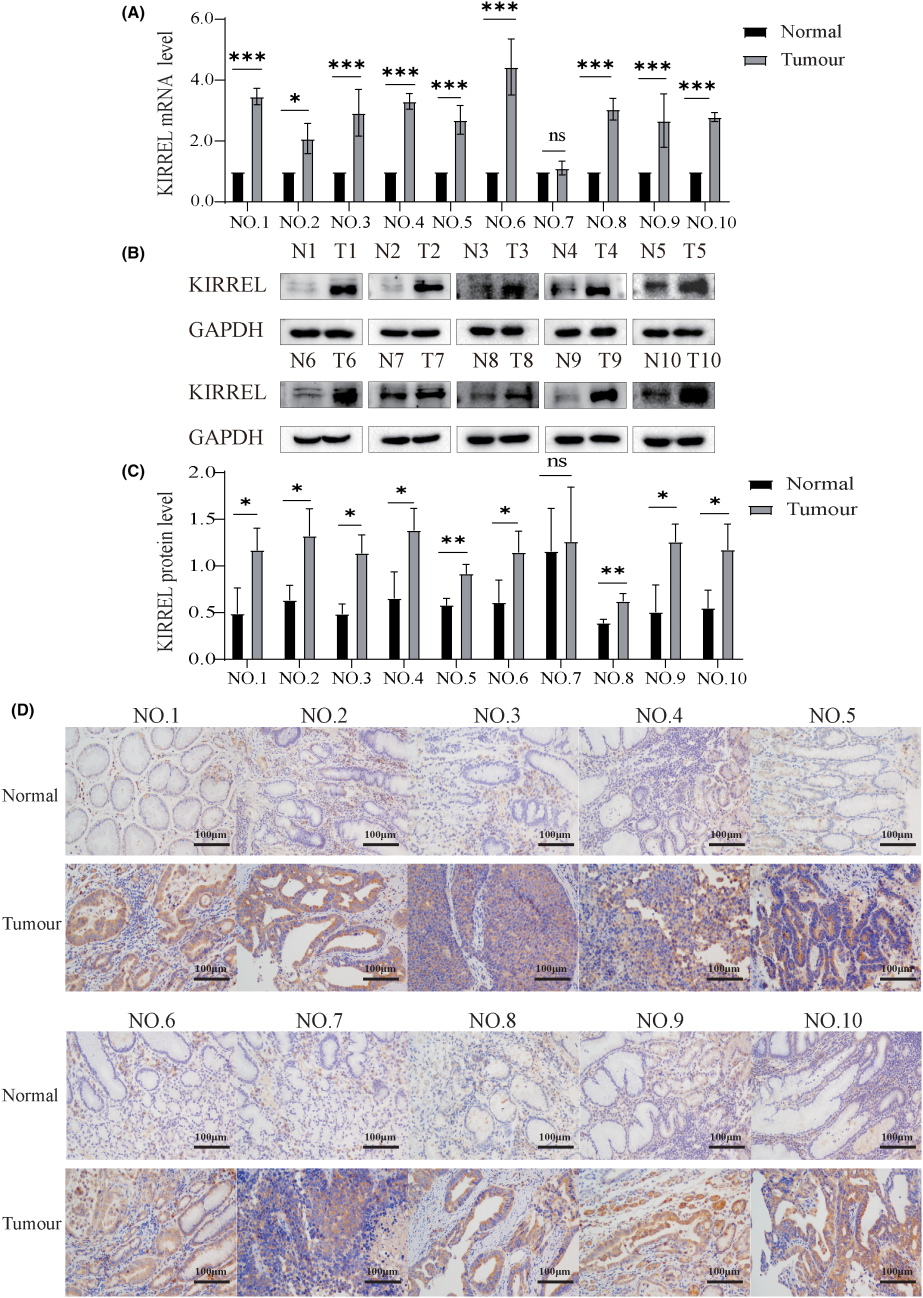
KIRREL expression is increased in GC tissues. (A) The expression of KIRREL mRNA in GC tissues was significantly higher than that in the adjacent tissues. (B, C) The expression of KIRREL protein in GC tissues was higher than that in the adjacent tissues. (D) The results of immunohistochemistry showed that the expression of KIRREL in tumour tissues was higher than that in the adjacent tissues; scale bar: 100 μm (sample size = 10; repetition = 3; *p < 0.05; **p < 0.01; ***p < 0.001).

Our previous studies have demonstrated that KIRREL expression is significantly elevated in GC compared with normal tissues by IHC and is associated with poor prognosis.^39^ In this study, we further verified the expression of KIRREL in GC tissues and adjacent tissues. Our qRT‐PCR results showed that the expression of KIRREL mRNA in tumour tissues was significantly higher than that in the adjacent tissues (Figure 1A). WB and IHC results also confirmed that the expression of KIRREL protein in GC tissues was significantly higher than that in adjacent tissues (Figure 1B–D).

### 3.2 Overexpression of KIRREL may play a role in GC cells through the PI3K/AKT signalling pathway

**FIGURE 2:**
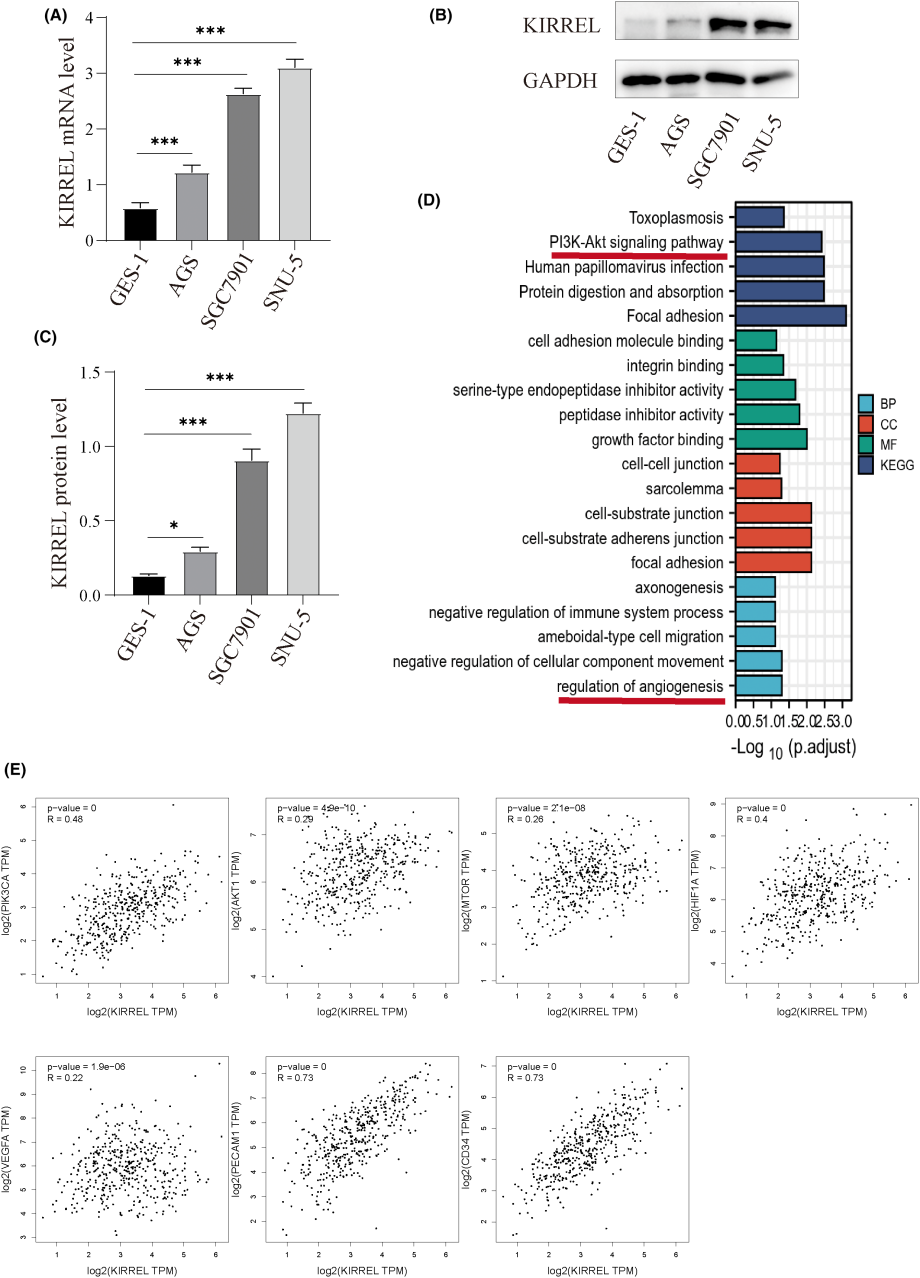
KIRREL expression in different GC cell lines and bioinformatics analysis. In three human GC cell lines SGC7901, SNU‐5 and AGS, the expression of mRNR (A) and encoded protein (B, C) of KIRREL was higher than that of normal gastric epithelial cell line GES‐1. (D) KEGG pathway and GO function (BP, CC and MF) enrichment analysis showed that KIRREL was enriched in the PI3K/AKT signalling pathway and angiogene‐related functions. (E) KIRREL was positively correlated with PIK3CA, AKT1, mTOR, HIF‐1α, VEGFA, PECAM1 and CD34 (repetition = 3; *p < 0.05; **p < 0.01; ***p < 0.001; ns, no significant difference).

Both qRT‐PCR and WB were used to detect KIRREL mRNA and protein expression levels in different GC cell lines. Compared with the normal gastric cell line GES‐1, the expression of KIRREL mRNA and protein was increased in the GC cell lines AGS, SGC7901 and SNU‐5, with the highest expression in SNU‐5 and the lowest expression in AGS (Figure 2A–C). Subsequently, we downloaded the GC gene expression data from the TCGA database and analysed them using R software to screen 219 genes with co‐expression relationships with KIRREL (|cor| > 0.7, *p* < 0.05). We performed an enrichment analysis for the top 50 genes with correlation coefficients and visualized the results (Figure 2D). Notably, KEGG and GO analyses showed that these genes were significantly enriched in the ‘PI3K/AKT signalling pathway’ and in the ‘regulation of angiogenesis’ function. Therefore, we hypothesized that the KIRREL gene may play a role in GC progression through the PI3K/AKT signalling pathway.

**FIGURE 3:**
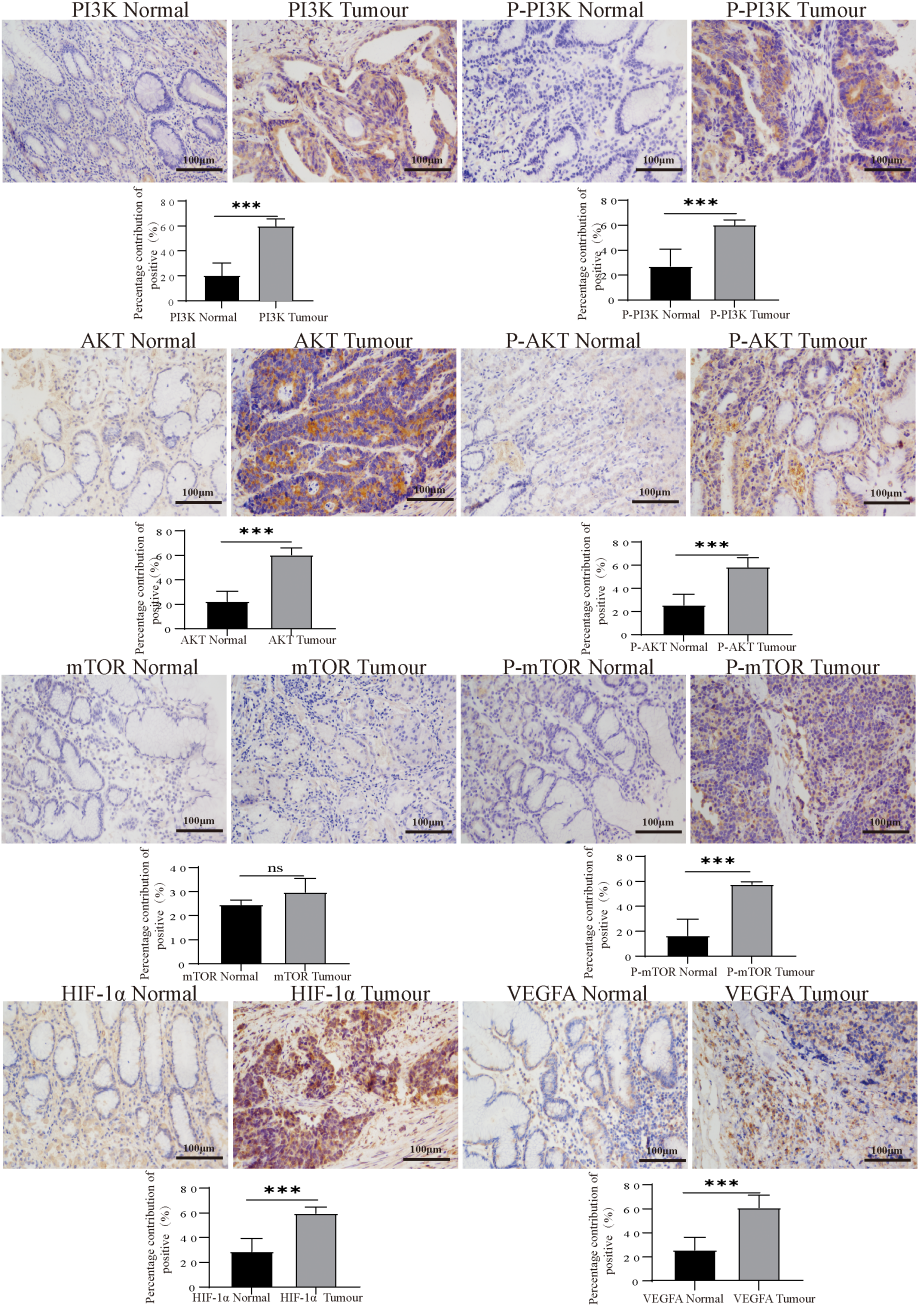
IHC results showed that the expressions of PI3K, P‐PI3K, AKT, P‐Akt, P‐mTOR, HIF‐1α and VEGF in human GC tissues were higher than those in the adjacent normal tissues (scale bar: 100 μm; sample size = 10; repetition = 3).

In addition, based on the GC gene expression data in the TCGA database, we analysed the correlation between KIRREL and some key genes in the PI3K/AKT signalling pathway using the GEPIA website. We found that the expression of PIK3CA, AKT1, mTOR, HIF‐1α, VEGFA, PECAM1 and CD34 was positively correlated with the KIRREL gene (Figure 2E). IHC results also showed that the expression of PI3K, P‐PI3K, AKT, P‐AKT, P‐mTOR, HIF‐1α and VEGFA in GC tissues was higher than that in the adjacent tissues (Figure 3).

### 3.3 KIRREL overexpression promoted the proliferation of GC cells

**FIGURE 4:**
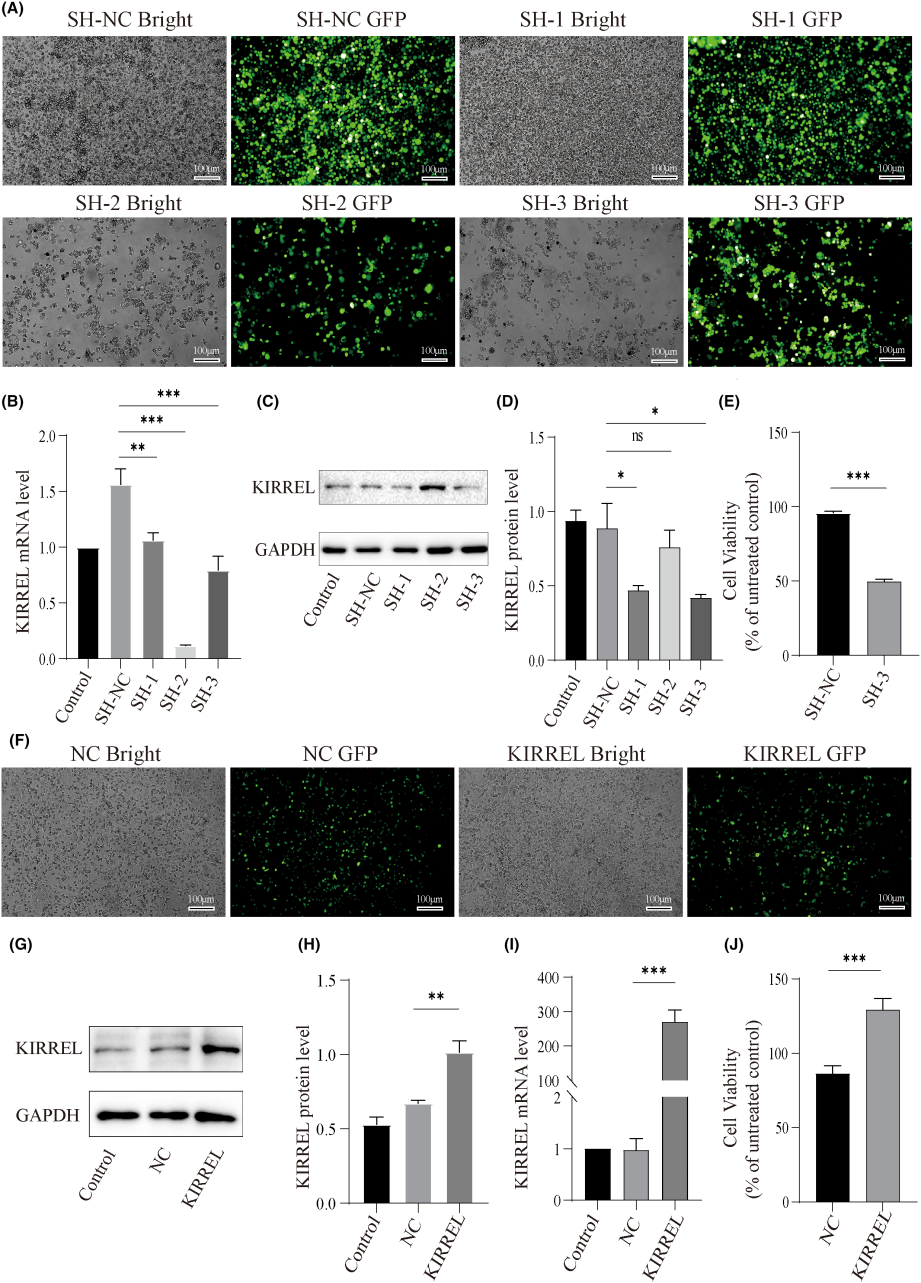
KIRREL overexpression promotes proliferation of GC cells. (A) The lentivirus transfection rate of SNU‐5 cells in each group was more than 80%; scale bar: 100 μm; Expression of KIRREL gene encoded protein (B, C) and KIRREL mRNA (D) in SNU‐5 cells transfected with lentivirus. (E) The results of CCK‐8 showed that the cell proliferation ability of SNU‐5 cell line was significantly reduced after KIRREL gene silencing compared with the control group. (F) The lentivirus transfection rate of AGS cells in each group was more than 80%; scale bar: 100 μm; Expression of KIRREL gene encoded protein (G, H) and KIRREL mRNA (I) in AGS cells transfected with lentivirus. (J) The CCK‐8 results showed that the proliferation of AGS cells was significantly enhanced after KIRREL gene overexpression compared with the control group (Control: blank control group; SH‐NC, NC: negative control group; SH‐1, SH‐2, SH‐3: Three types of knockdown groups; KIRREL: overexpression group; repetition = 3; *p < 0.05; **p < 0.01; ***p < 0.001; ns, no significant difference).

A gene‐knockout assay was used to investigate the biological effects of KIRREL in GC cells. To screen out the shRNA with the best knockout effect of the target gene, we infected the SNU‐5 cell line (Cell line with highest KIRREL protein expression) with lentiviruses of SH‐NC, SH‐1, SH‐2 and SH‐3 and recorded the lentivirus infection by fluorescence microscopy (Figure 4A). In addition, qRT‐PCR results indicated that the knockdown effects of SH‐1, SH‐2 and SH‐3 were statistically different (Figure 4B), but WB results showed that the SH‐3 knockdown effect had the best results (Figure 4C,D), so SH‐3 was used in the subsequent experiments.

The CCK‐8 assay was used to detect the activity and proliferation of GC cells. We found that the activity of KIRREL‐knockdown SNU‐5 cells decreased after 48 h of culture (Figure 4E). As mentioned above, we studied three GC cell lines and found that the expression of KIRREL was the least significant in the AGS cell line. Therefore, we overexpressed KIRREL in AGS (Figure 4F–I), and the CCK‐8 test results showed that the proliferative ability of AGS cells was significantly increased after KIRREL overexpression treatment (Figure 4J). These results suggest that KIRREL overexpression enhances the activity and proliferation of GC cells.

### 3.4 Silencing of the KIRREL gene induced G0/G1 arrest in GC cells and inhibited tumour angiogenesis

**FIGURE 5:**
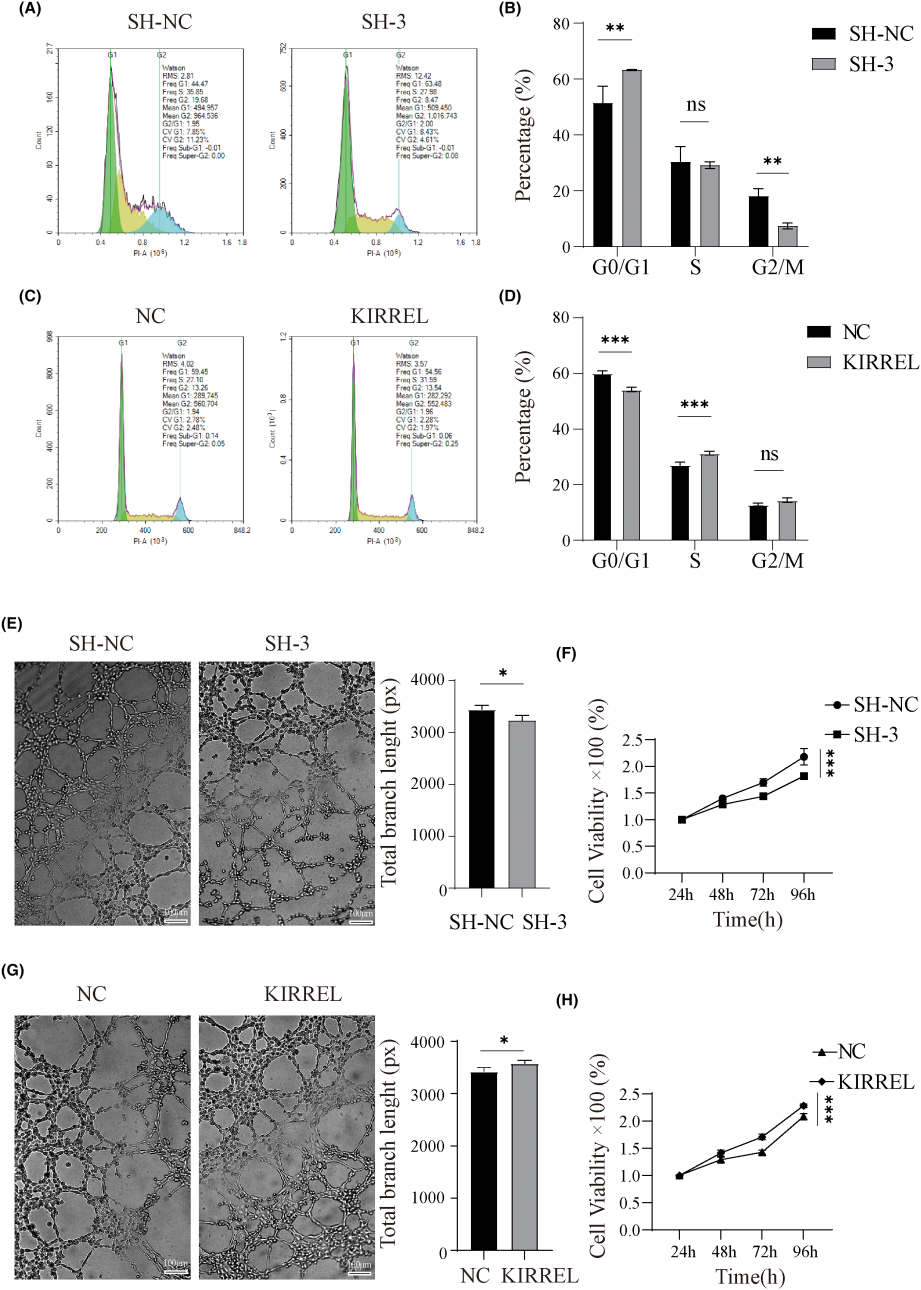
KIRREL gene silencing induced G0/G1 arrest and inhibited tumour angiogenesis in GC cells. (A, B) The flow cytometry was used to detect the cell cycle distribution of SNU‐5 cells after KIRREL gene knockdown. (C, D) Flow cytometry was used to detect the cell cycle distribution of SNU‐5 cells after KIRREL gene overexpression. (E) The results of tube formation assay showed that the ability of angiogenesis after KIRREL gene knockdown was significantly reduced compared with the SH‐NC group; scale bar: 100 μm. (F) The viability of HUVECs was examined by CCK8. (G) The angiogenesis ability of KIRREL gene overexpression was significantly enhanced compared with control group; scale bar: 100 μm. (H) The viability of HUVECs was examined by CCK8 (repetition = 3; *p < 0.05; **p < 0.01; ***p < 0.001; ns, no significant difference).

The cell cycle was detected by flow cytometry in the SNU‐5 knockdown, AGS overexpression and control groups. In the cell cycle of the SNU‐5 cell line, the proportion of cells in the G0/G1 phase in the SH‐NC group was the lowest (44.47%), whereas the proportion of cells in the G0/G1 phase in the SH‐3 group was the highest (63.48%). No significant difference was observed in the proportion of the S phase between the two groups (Figure 5A,B). The opposite result was observed in the G0/G1 phase of the AGS cell line (Figure 5C,D), and the proportion of cells in the S phase increased. These results suggest that KIRREL knockdown can induce G0/G1 arrest in GC cells and overexpression of KIRREL can reverse this effect.

Tumour angiogenesis is closely related to tumour growth, progression and metastasis.^40^, ^41^ Therefore, we also performed a HUVEC tube formation assay and CCK‐8 assay to explore the role of KIRREL in angiogenesis. The medium supernatants of these cells were collected and used to culture HUVECs. The results showed that in the tube formation assay of SNU‐5 cell line, the total vessel branch length in the SH‐3 group (mean 3231px, SD 97.08px) was significantly shorter than that in the SH‐NC group (mean 3448px, SD 77.57), and the viability of the HUVECs in the SH‐3 group was also significantly poorer than that in the SH‐NC group (Figure 5E,F). However, in the tube formation assay of AGS cell lines, the total vessel branch length was significantly longer in the KIRREL group (mean 3583px, SD 55.43px) than in the NC group (mean 3430px, SD 69.87px), and the viability of HUVECs was also significantly higher in the KIRREL group than in the NC group (Figure 5G,H). These results suggest that high KIRREL expression promotes angiogenesis.

### 3.5 KIRREL overexpression may promote the proliferation and angiogenesis of GC cells by activating the PI3K/AKT/mTOR signalling pathway

**FIGURE 6:**
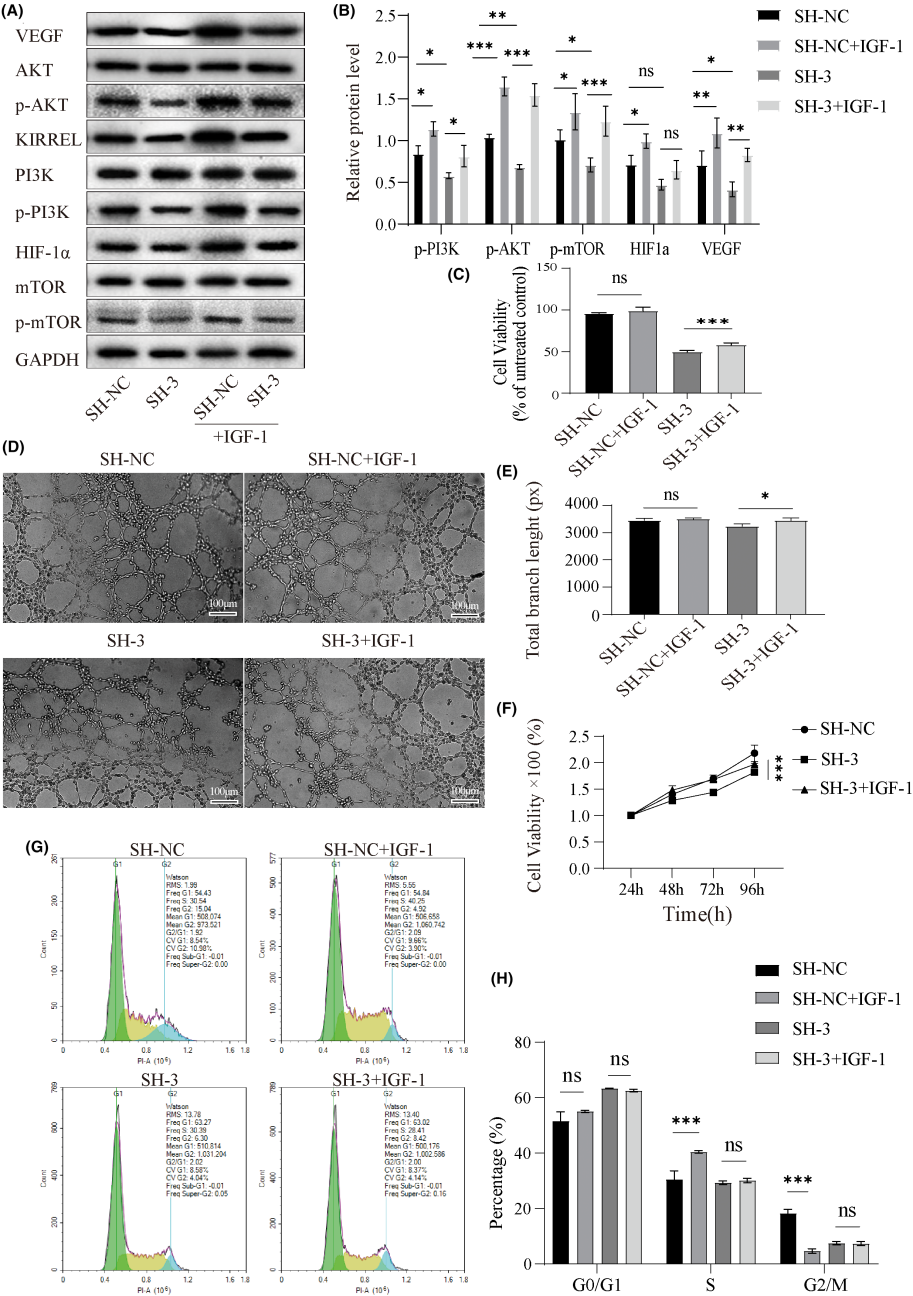
AKT agonist IGF‐1 reversed KIRREL silencing induced PI3K/AKT/mTOR pathway inhibition and GC cell proliferation and angiogenesis. (A, B) After IGF‐1 (50 ng/mL) was added to lentivirus transfected SNU‐5 cells, the expressions of related proteins in the PI3K/AKT/mTOR pathway, HIF‐1α and VEGF were increased (C) The CCK‐8 results showed that the proliferation ability of GC cells was also enhanced. (D, E) Tube formation assay showed that the ability of angiogenesis of SNU‐5 cells transfected with lentivirus was also significantly enhanced when IGF‐1 was added; scale bar: 100 μm. (F) The viability of HUVECs was examined by CCK8. (G, H) The flow cytometry showed that IGF‐1 had no significant effect on the cycle distribution of SNU‐5 cells transfected with lentivirus (repetition = 3; *p < 0.05; **p < 0.01; ***p < 0.001; ns, no significant difference).

Based on the results of the bioinformatics analysis, we further investigated the potential mechanism of KIRREL in the PI3K/AKT/mTOR signalling pathway. WB results of the SNU‐5 group showed that KIRREL‐knockdown in the SH‐3 group resulted in significantly lower phosphorylation of PI3K, AKT and mTOR than in the SH‐NC group, while total PI3K, AKT and mTOR proteins were not affected (Figure 6A,B). This suggests that PI3K/AKT signalling was not activated in the KIRREL‐knockdown SNU‐5 cells. We also detected a decreased expression of VEGF and HIF‐1α, both of which are factors associated with angiogenesis (Figure 6A,B). Subsequently, the AKT pathway agonist insulin‐like growth factor 1 (IGF‐1) was applied to the SH‐3 and SH‐NC groups, and sustained activation of the PI3K/AKT pathway was observed (Figure 6A,B). The inhibition of SNU‐5 cell proliferation (Figure 6C) and the viability and angiogenesis ability of HUVECs (Figure 6D–F) induced by KIRREL knockdown were reversed by IGF‐1 treatment (total vessel branch length in the SH‐3 group increased from a mean of 3231px, SD 97.08px, before treatment to a mean of 3349px, SD 94.03px). However, IGF‐1 was not effective in reversing the G0/G1 arrest induced by KIRREL knockdown (Figure 6G,H).

**FIGURE 7:**
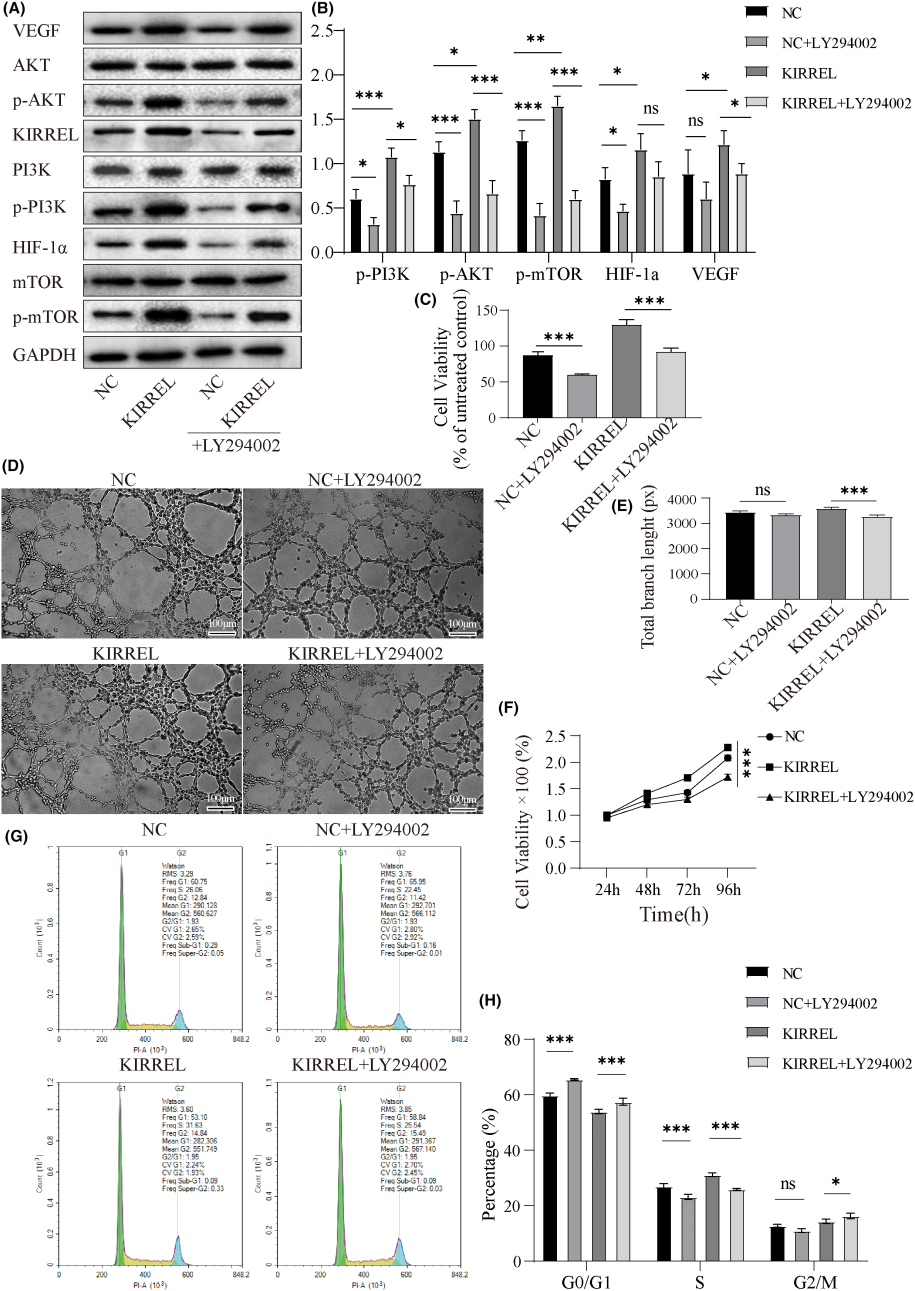
AKT inhibitor LY294002 reversed KIRREL overexpression induced the PI3K/AKT/mTOR pathway activation and GC cell proliferation and angiogenesis. (A, B) After LY294002 (5 μM) was added to AGS cells transfected with lentivirus, the expressions of related proteins in the PI3K/AKT/mTOR pathway, HIF‐1α and VEGF were decreased. (C) The CCK‐8 results showed that the proliferation ability of GC cells was also weakened. (D, E) Tube formation assay showed that the ability of angiogenesis in AGS cells transfected with lentivirus was also significantly reduced after LY294002 was added; scale bar: 100 μm. (F) The viability of HUVECs was examined by CCK8. (G, H) The results of flow cytometry showed that the S‐cycle of AGS cells transfected with lentivirus was shortened, while the G0/G1 and G2/M cycles were prolonged after LY294002 was added (repetition = 3; *p < 0.05; **p < 0.01; ***p < 0.001; ns, no significant difference).

In addition, we applied the AKT pathway inhibitor LY294002 to the AGS cells in each group, and the results showed that the expression of HIF‐1α and the phosphorylation of PI3K, AKT and mTOR increased significantly with KIRREL overexpression (Figure 7A,B). Treatment with LY294002 reduced the expression of HIF‐1α and phosphorylation of PI3K, AKT and mTOR, and reversed the promotion of cell proliferation (Figure 7C) and the viability and angiogenesis ability of HUVECs induced (total vessel branch length in the KIRREL group was reduced from a mean of 3583 px, SD 55.43 px, before treatment to a mean of 3264 px, SD 65.83 px) by KIRREL overexpression in the AGS cell line (Figure 7D–F). LY294002 treatment also increased G0/G1 arrest (Figure 7G,H). These results suggest that the pro‐cancer effect of KIRREL gene may be mediated by the PI3K/AKT/mTOR signalling pathway.

## 4 DISCUSSION

GC is the second most common cause of cancer‐related deaths worldwide.^42^ Owing to its high intra‐tumour and inter‐tumour heterogeneity, the overall treatment response of GC remains unsatisfactory.^43^ Angiogenesis plays an important role in the tumour microenvironment. Pathological abnormalities and dysfunction of tumour vessels often lead to hypoxia and an increased risk of metastasis.^44^ Therefore, angiogenesis targeting is a promising therapeutic strategy.

KIRREL is a cell adhesion molecule that was first discovered in gene database screening and was named NEPH1 because of its structural similarity to nephrin in the human kidney.^32^ However, the role of KIRREL in tumours continues to not be well understood. Chen et al. reported that KIRREL is overexpressed in breast cancer and that high KIRREL expression can be used as an independent predictor of poor prognosis in patients with breast cancer.^37^ In thin melanomas (Breslow thickness ≤ 1 mm), high protein expression of KIRREL was significantly associated with both poor RFS and reduced MSS.^38^ Wang et al. reported that KIRREL directly bound to SAV1 and activated the Hippo tumour suppressor pathway thereby inhibiting the growth of colon cancer cells in vitro and in vivo.^45^ Gimenez‐Xavier et al. found that KIRREL had a significantly higher mutation frequency in EBC1‐R, a human lung squamous cell carcinoma cell line sensitive to erlotinib.^46^ Other researchers have found that differentially methylated sites in osteosarcoma are associated with up‐regulation of the expression of genes such as KIRREL, which may be contributing to osteosarcoma progression by promoting cell proliferation and metastasis.^47^ In a gene fusion study in benign fibrous histiocytomas, KIRREL was found to be chimeric with PRKCA thus confirming the involvement of KIRREL and PRKCA in the development of benign fibrous histiocytomas.^48^ Although our previous studies have demonstrated that KIRREL is overexpressed in human GC tissues and is associated with poor prognosis, its mechanism of action remains unclear. In this study, we used bioinformatics analyses to screen KIRREL‐related functions and pathways, including ‘negative regulation of the immune system process’, ‘regulation of angiogenesis function’, ‘focal adhesion’ and ‘PI3K/AKT signalling pathway’, all of which play key roles in cancer progression.^49^, ^50^ Many studies have demonstrated that activation of the PI3K/AKT/mTOR signalling pathway can promote cell proliferation and resist cell cycle arrest.^51^, ^52^, ^53^ These analyses suggest that KIRREL may be associated with the proliferation and growth of GC cells. In the study of PI3K/AKT/mTOR pathway‐related proteins in GC tissues, one found that PI3K AKT, P‐Akt, P‐mTOR, HIF‐1α and VEGF were significantly higher expressed in GC than in paracancerous tissues, whereas there was no difference in mTOR, which was consistent with our findings.^54^, ^55^, ^56^ Furthermore, KIRREL knockdown inhibited the viability of GC cells and induced G0/G1 arrest. After the addition of IGF‐1, an AKT pathway activator, KIRREL knockdown‐induced cell proliferation was significantly reversed, but G0/G1 arrest was not significantly altered. This may be due to the potential autocrine activity of IGF‐1 in tumour cells^57^ leading to insignificant activation of exogenous IGF‐1, or due to the low expression of IGF‐1R in the SNU‐5 cell line^58^ leading to insignificant activation of exogenous IGF‐1.

It is worth mentioning that our study also found that KIRREL was involved in the regulation of angiogenesis. The PI3K/AKT signalling pathway, HIF‐1α and VEGFA are closely associated with angiogenesis. The activated PI3K/AKT pathway has been reported to promote the production of VEGF independently or may be dependent on HIF‐1, and the PI3K/AKT pathway can also regulate angiogenic factors such as angiopoietin and carbon monoxide.^59^, ^60^, ^61^ Stable HIF‐1α binding to HIF‐1β can also induce the expression of angiogenic factors VEGF, PDGF‐B, Ang‐1 and Ang‐2,^62^, ^63^ while HIF‐1α deletion can reduce the angiogenic behaviour of endothelial cells.^64^ The HUVEC tube formation assay results confirmed that KIRREL overexpression promotes angiogenesis, and the WB assay results confirmed that KIRREL overexpression induces an increase in HIF‐1α and VEGF expression and activation of the PI3K/AKT/mTOR signalling pathway. In addition, our results showed that the AKT inhibitor LY294002 significantly reverses AKT phosphorylation, and downregulates HIF‐1α and VEGF expression and KIRREL‐mediated angiogenesis. Of course, there are still many shortcomings in our study, whether KIRREL has an effect on the malignant phenotype of GC in vivo we have not yet studied, as well as the design of small‐molecule inhibitors targeting KIRREL, which are the direction and content of the next step of our research.

## 5 CONCLUSION

In conclusion, our study confirms the overexpression of KIRREL in GC tissues and cell lines and provides evidence that KIRREL promotes GC proliferation and angiogenesis by activating the PI3K/AKT/mTOR pathway. We revealed that KIRREL may be an effective target for the treatment of GC, providing an important reference for the development of targeted drugs in the future.

## AUTHOR CONTRIBUTIONS

**Tao Wang:** Data curation (lead); formal analysis (lead); methodology (supporting); software (lead); visualization (lead); writing – original draft (lead); writing – review and editing (lead). **Shuo Chen:** Methodology (equal); project administration (equal). **Ziliang Wang:** Writing – review and editing (equal). **Siyu Li:** Data curation (equal). **Xichang Fei:** Data curation (equal). **Tong Wang:** Data curation (equal). **Mingjun Zhang:** Conceptualization (lead); resources (lead); supervision (lead).

## FUNDING INFORMATION

This research was supported by Natural Science Foundation of Anhui (1908085MH262).

## CONFLICT OF INTEREST STATEMENT

The authors declare that they have no conflicts of interest.

## CONSENT FOR PUBLICATION

All authors have reviewed and confirmed the final version of the manuscript and have agreed to its publication.

## Data Availability

The original contributions presented in the study are included in the article; further inquiries can be directed to the corresponding author. The datasets analysed during the current study are available in the TCGA repository (https://portal.gdc.cancer.gov/).
